# Diagnostic Utility of Acetic Acid-induced Acetowhite Change in Suspected Penile Intraepithelial Neoplasia

**DOI:** 10.7759/cureus.107021

**Published:** 2026-04-14

**Authors:** Jawwad M Haider, Syed Kamran

**Affiliations:** 1 Urology, St George's Hospital, London, GBR; 2 Urology, City St George's, University of London, London, GBR

**Keywords:** biopsy, penile intraepithelial neoplasia (pein), penile lesion, urologic cancer, visual inspection with acetic acid

## Abstract

Introduction: Penile intraepithelial neoplasia (PeIN) is a premalignant lesion with clinical features that often overlap with benign inflammatory dermatoses. Although biopsy remains the diagnostic gold standard, topical acetic acid application producing an acetowhite reaction is sometimes used to aid clinical assessment. This study evaluated the diagnostic utility of acetic acid-induced acetowhite change in identifying penile dysplasia or PeIN.

Methods: A retrospective observational cohort study was conducted at a tertiary penile cancer referral centre. Consecutive patients referred with suspicious erythematous penile lesions between September 2022 and September 2025 who underwent both topical acetic acid application and penile biopsy were included. Acetowhite responses were categorised as negative, mildly positive, or strongly positive based on contemporaneous clinical documentation. Histopathological diagnosis from biopsy specimens served as the reference standard. The association between acetowhite response and histological outcome was assessed using chi-squared analysis.

Results: Fifty-two patients were included. Histopathology demonstrated benign findings in 34 patients, PeIN in 10, mild dysplasia in 1, and moderate-severe dysplasia in 7. Acetowhite change was observed across both benign and dysplastic lesions. No significant association was identified between the presence or degree of acetowhite change and histologically confirmed dysplasia or PeIN (p = 0.686).

Conclusion: Acetic acid-induced acetowhite change did not reliably predict the presence of penile dysplasia or PeIN. These findings suggest that acetic acid testing has limited diagnostic utility in the assessment of suspicious penile lesions and should not influence decisions regarding biopsy, which remains essential for accurate diagnosis regardless of the response to acetic acid application.

## Introduction

Penile intraepithelial neoplasia (PeIN) is a recognised premalignant epithelial lesion of the penis and the precursor to invasive penile squamous cell carcinoma (PSCC) [1]. PeIN includes a spectrum of histological subtypes that fall into human papillomavirus (HPV)-associated and HPV-independent pathways, with differentiated (non-HPV) and basaloid/warty (HPV-associated) phenotypes [2]. Clinically, these lesions may present as persistent erythematous patches, plaques, or subtle areas of erythema on the glans, inner prepuce, or sulcus and are frequently difficult to distinguish from benign inflammatory or infectious dermatological processes based on visual inspection alone [3].

Because of the clinical overlap between premalignant PeIN and benign penile dermatoses, histopathological confirmation via biopsy remains the diagnostic gold standard to establish PeIN and exclude invasive carcinoma [4]. However, clinicians often use adjunctive techniques to aid visual assessment. One such technique is the application of topical acetic acid (typically 3-5%) with subsequent assessment for an "acetowhite" reaction, a practice borrowed from colposcopy in cervical dysplasia, where acetowhitening can highlight abnormal squamous epithelium [5].

In cervical screening, the acetowhite change represents reversible protein coagulation in dysplastic epithelium and is used as a marker during colposcopy, albeit with known limitations in specificity and predictive value [5]. Extrapolation of this principle to the penile epithelium has led some clinicians to apply acetic acid in the clinic to improve lesion detection and guide biopsies in patients presenting with suspected dysplasia or PeIN [6]. Early genital dermatology and penoscopy studies demonstrated that acetowhite change frequently occurs in benign or inflammatory conditions and does not reliably correspond to dysplasia or HPV infection, which raises questions about its specificity as a diagnostic tool in the male genital setting [7].

Despite these concerns and the limited evidence base specifically addressing PeIN, acetic acid testing continues to be used in some tertiary penile cancer referral centres as part of the clinical assessment of men with suspicious penile lesions [8]. Given its routine use in clinical practice and implications for biopsy strategies and patient counselling, it is critical to evaluate whether the acetowhite change correlates with histologically confirmed dysplasia. In this retrospective cohort study of consecutively referred patients with suspected PeIN, we analyse the association between clinical acetic acid test results and biopsy outcomes to assess the diagnostic utility of acetowhite change in suspected penile dysplasia and subsequently whether acetic acid application should be used to influence clinical decisions regarding proceeding to biopsy.

## Materials and methods

Study design and setting 

This was a retrospective observational cohort study conducted at St George's Hospital, London, a tertiary referral centre for penile cancer. The study evaluated the diagnostic utility of topical acetic acid application in patients referred with suspected PeIN or penile dysplasia.

Patient selection 

All consecutive patients attending the specialist penile cancer clinic over the study period from 01/09/2022 to 01/09/2025 who were referred with a suspicious erythematous penile lesion and subsequently underwent both clinical acetic acid application and penile biopsy were included. Patients were identified by retrospective review of clinic records. There were no exclusions based on age, lesion morphology, or biopsy result. In total, 52 patients met the inclusion criteria. 

Acetic acid application and clinical assessment 

As part of routine clinical practice at the centre, topical acetic acid (5%) was applied to the affected area of penile epithelium for two minutes during clinic assessment. Following application, lesions were assessed visually by the examining clinician, and observed acetowhite responses were descriptively documented. Clinicians were not blinded to prior available information on patients. Responses were categorised according to the observed acetowhite response as negative (no visible whitening), mildly positive, or strongly positive based on the description recorded. Any acetowhite changes described using the terms "some", "mild" or "slight" were categorised as mildly positive, while acetowhite changes categorically recorded as positive or described using terms "strong" or "extensive" were categorised as strongly positive. The acetic acid result was documented contemporaneously in the clinic record and was used for clinical planning, but did not replace biopsy.

Histopathological assessment 

All patients underwent a penile biopsy following clinical assessment. Histopathological analysis was performed by specialist uropathologists in accordance with standard diagnostic criteria. Biopsy results were classified as follows: (1) benign, (2) PeIN, (3) mild dysplasia, or (4) moderate-severe dysplasia. For statistical analysis, biopsy findings were considered the reference (gold) standard for diagnosis.

Statistical analysis

The association between acetic acid staining result and biopsy outcome was assessed using a chi-squared (χ²) test. Expected frequencies were calculated to ensure appropriateness of the test and confirmed to be sufficient. Statistical significance was defined as a p-value <0.05. All analyses were performed using spreadsheet-based statistical tools. 

Ethics

This study was conducted as a retrospective service evaluation of routine clinical practice. All data were anonymised prior to analysis, and no patient-identifiable information was retained.

## Results

A total of 52 consecutive patients referred to the tertiary penile cancer clinic with suspected penile dysplasia, or PeIN, were included. Patients had a mean age of 63.4 ± 13.9 years (range: 24-91 years). Detailed demographic and clinical characteristics of the study population are presented in Table 1.

**Table 1:** Demographic findings

Demographic findings	Categories	Frequency, N (%)
Age at biopsy	21-30	3 (5.8%)
	31-40	1 (1.9%)
	41-50	3 (5.8%)
	51-60	8 (15.4%)
	61-70	19 (36.5%)
	71-80	15 (28.8%)
	81-90	2 (3.8%)
	91-100	1 (1.9%)
Smoking status	Smoker	15 (28.8%)
	Non-smoker	37 (71.1%)
Immunosuppressed	Yes	7 (13.5%)
	No	45 (86.5%)
History of phimosis	Yes	14 (26.9%)
	No	38 (73.1%)
History of sexually transmitted infections	Yes	6 (11.5%)
	No	46 (88.5%)

All patients underwent clinical assessment with topical acetic acid application, followed by penile biopsy. There was no missing data for acetic acid test results or histopathological outcomes. The distribution of biopsy-confirmed diagnoses is summarised in Table 2.

**Table 2:** Histopathological outcomes of penile biopsy (n = 52) PeIN: Penile intraepithelial neoplasia

Histopathological diagnosis	Number of patients, N (%)
Benign	34 (65.4%)
PeIN	10 (19.2%)
Mild dysplasia	1 (1.9%)
Moderate-severe dysplasia	7 (13.5%)

Benign pathology constituted the majority of cases. Premalignant lesions (PeIN and dysplasia combined) accounted for 18 (34.6%) cases, indicating that a substantial proportion of clinically suspicious lesions were non-neoplastic despite referral to a specialist centre. Benign findings included acute inflammation, chronic inflammation, lichenoid dermatitis, atypia, and condyloma acuminatum.

For analysis, biopsy outcomes were grouped into benign and dysplasia/PeIN categories. The relationship between acetic acid staining response and histopathological diagnosis is shown in Table 3.

**Table 3:** Association between acetic acid staining result and histopathological outcome Statistical test: Chi-squared (χ²) test of independence

Acetic acid stain result	Benign, N (%)	Dysplasia/PeIN, N (%)	Total, N (%)	χ² value	p-value
Negative	12 (23.1%)	5 (9.6%)	17 (32.7%)		
Mildly positive	11 (21.2%)	5 (9.6%)	16 (30.8%)		
Strongly positive	11 (21.2%)	8 (15.4%)	19 (36.5%)		
Total	34 (65.4%)	18 (34.6%)	52 (100%)	0.754	0.686

Acetowhite change was observed across both benign and dysplastic lesions, with no consistent trend indicating increased likelihood of dysplasia with stronger staining. Notably, five patients with dysplasia/PeIN demonstrated no acetowhite response, while a comparable proportion of benign lesions exhibited strong acetowhitening. Chi-squared analysis demonstrated no statistically significant association between acetic acid staining category and biopsy outcome (χ² = 0.754, p = 0.686), indicating that acetowhite response does not reliably predict the presence of premalignant disease.

## Discussion

In this retrospective consecutive cohort study of 52 patients referred with suspected penile dysplasia, we found no statistically significant association between acetowhite change following topical acetic acid application and histologically confirmed PeIN or dysplasia. The chi-squared analysis demonstrated a non-significant result (χ² = 0.754, p = 0.686), indicating that the presence or degree of acetowhite change did not reliably predict underlying dysplasia on biopsy.

These findings challenge the assumed diagnostic utility of acetic acid testing in the assessment of suspicious penile lesions. Although acetic acid application is widely used in some specialist centres to aid lesion detection or delineation [9], its use in penile pathology is largely extrapolated from cervical colposcopy, where acetowhitening reflects reversible coagulation of nuclear proteins in dysplastic epithelium. Importantly, even in the cervical setting, visual inspection with acetic acid is known to have limited specificity, with acetowhite change frequently observed in benign or inflammatory epithelium [10].

The limited specificity of acetowhite change in male genital skin has been previously reported. Penoscopic studies evaluating acetowhite lesions in men have demonstrated high false-positive rates, with many acetowhite areas corresponding to benign inflammatory changes rather than HPV infection or dysplasia [6]. These findings suggest that acetowhite change may represent a non-specific epithelial response rather than a marker of premalignant disease, particularly in the penile epithelium, which is frequently affected by inflammation, irritation, and prior topical treatments.

Despite this, acetic acid testing continues to be used in some tertiary penile cancer referral clinics, including our own, to inform biopsy strategy and clinical planning. Our results suggest that reliance on acetowhite change may be misleading and could potentially contribute to unnecessary biopsies or false reassurance. Current international guidelines emphasise that persistent or suspicious penile lesions require histological assessment, as clinical appearance alone is insufficient to reliably distinguish PeIN from benign dermatoses [11]. The findings of this study support this guidance and suggest that acetic acid testing should not influence decisions regarding whether to biopsy.

There are several limitations to this study that should be acknowledged. The retrospective design limits control over documentation quality and inter-observer variability in grading the acetowhite response. Additionally, the sample size, while representative of a specialist referral practice, may limit the detection of small effect sizes. Finally, categorisation of acetowhite change was subjective and reflects real-world clinical practice rather than standardised image-based assessment.

## Conclusions

In this study, topical acetic acid application and the presence or degree of acetowhite change did not demonstrate any meaningful association with histologically confirmed PeIN or dysplasia. Acetowhite responses were frequently observed in both benign and premalignant lesions, while a notable proportion of dysplastic cases showed no visible reaction, highlighting the test's poor sensitivity and specificity. These findings suggest that acetic acid testing lacks diagnostic reliability as an adjunct in the assessment of suspicious penile lesions. Although these results indicate that routine use of acetic acid in this context should be reconsidered, with greater emphasis placed on thorough clinical evaluation and timely histopathological confirmation, larger-scale studies with more objective techniques for quantifying acetowhite changes are needed.
